# Alpha Calcitonin Gene-Related Peptide Increases Cerebral Vessel Diameter in Animal Models of Subarachnoid Hemorrhage: A Systematic Review and Meta-analysis

**DOI:** 10.3389/fneur.2017.00357

**Published:** 2017-07-25

**Authors:** Liam M. C. Flynn, Caroline J. Begg, Malcolm R. Macleod, Peter J. D. Andrews

**Affiliations:** ^1^Centre for Clinical Brain Sciences, University of Edinburgh, Edinburgh, United Kingdom; ^2^Emergency Department, Edinburgh Royal Infirmary, Edinburgh, United Kingdom

**Keywords:** calcitonin gene-related peptide, CGRP, cerebral vasospasm, delayed cerebral ischemia, subarachnoid hemorrhage

## Abstract

Delayed cerebral ischemia (DCI) is a life-threatening complication after subarachnoid hemorrhage. There is a strong association between cerebral vessel narrowing and DCI. Alpha calcitonin gene-related peptide (αCGRP) is a potent vasodilator, which may be effective at reducing cerebral vessel narrowing after subarachnoid hemorrhage (SAH). Here, we report a meta-analysis of data from nine *in vivo* animal studies identified in a systematic review in which αCGRP was administered in SAH models. Our primary outcome was change in cerebral vessel diameter and the secondary outcome was change in neurobehavioral scores. There was a 40.8 ± 8.2% increase in cerebral vessel diameter in those animals treated with αCGRP compared with controls (*p* < 0.0005, 95% CI 23.7–57.9). Neurobehavioral scores were reported in four publications and showed a standardized mean difference of 1.31 in favor of αCGRP (CI −0.49 to 3.12). We conclude that αCGRP reduces cerebral vessel narrowing seen after SAH in animal studies but note that there is insufficient evidence to determine its effect on functional outcomes.

## Introduction

Aneurysmal subarachnoid hemorrhage is a significant cause of morbidity and mortality worldwide with an annual incidence of 9 per 100,000 person years (1–3). 27% of all stroke-related potential years of life lost before the age of 65 years are attributable to subarachnoid hemorrhage (SAH), and the average age at first onset of SAH is 55 years (4–6). Of the 85–90% of patients who survive to reach hospital, up to 42% will die within 1 month of the SAH and 20% remain dependent on others for activities of daily living (6, 7).

Despite successful treatment of the ruptured aneurysm and reduction of the risk of rebleeding, approximately 30% of treated patients develop focal neurological or cognitive deficits conventionally attributed to delayed cerebral ischemia (DCI) (8–11). A strong association exists between cerebral vessel narrowing and DCI, and it has been suggested that treating vessel narrowing after SAH may improve outcomes (12).

Alpha calcitonin gene-related peptide (αCGRP) is an endogenous neuropeptide and powerful vasodilator. αCGRP exerts its relaxing properties through nitric oxide and endothelium-dependent or endothelin-independent pathways (13). Endogenous αCGRP appears to be released and subsequently depleted in response to cerebral vasoconstriction after SAH, leading to the suggestion that exogenous αCGRP may be beneficial in managing DCI (14–16). Several animal studies and three human trials have investigated the effect of αCGRP on cerebral arteries after SAH with a view to administering αCGRP as a treatment for DCI (17–40). However, there has been no systematic summary assessing the efficacy of αCGRP in reducing cerebral vasospasm in animal models.

Here, we use this approach to summarize data from publications reporting *in vivo* animal studies that investigated the effects of αCGRP after experimental SAH.

## Methods

The study protocol has been published elsewhere and is available open access but a brief description is given below (41).

### Search Strategy and Study Selection

In January 2015, we searched two electronic databases (MEDLINE *via* PubMed Central, and EMBASE *via* OvidSP) using the key words “alpha calcitonin gene-related peptide,” “αCGRP,” and “subarachnoid hemorrhage” in combination using the Boolean operator [AND]. The search was restricted to “other animals.” Two investigators (Liam M. C. Flynn and Caroline J. Begg) independently screened the abstracts and titles to identify those that met our inclusion criteria. Any differences were resolved by discussion with a third reviewer (Peter J. D. Andrews). We included *in vivo* animal studies describing the effect of αCGRP in animal models of SAH where outcome was reported as a change in arterial diameter and the articles were published in English. We also included studies which examined *in vivo* SAH and αCGRP administration with postmortem *in vitro* measurements of artery diameter.

The above search was repeated in July 2017 using the same methods. No further studies meeting eligibility criteria were identified.

### Data Extraction

Two investigators independently extracted data relating to species of animal and weight; method of inducing SAH (single injection, double injection, or clot placement); whether the basilar artery, middle cerebral artery, anterior cerebral artery, or internal carotid artery were measured; the method of measurement (angiography, *in vitro* measurement or direct *in vivo* visualization); anesthetic agent used; dose of αCGRP and time of administration from SAH; reporting and method of randomization; reporting and method of blinding; animal welfare guideline statement; statement of sample size calculation; whether there was a statement of potential conflicts of interest and the use of animals with comorbidities. Study quality was assessed using the CAMARADES 10-item quality checklist (42). One point was awarded for each of (1) publication in a peer-reviewed journal, (2) statement of control temperature, (3) randomized intervention allocation, (4) intervention allocation concealment, (5) blinded assessment of outcome, (6) avoidance of anesthetics with marked intrinsic neuroprotective properties (ketamine), (7) statement of *a priori* sample size collection, (8) statement of compliance with regulatory requirements, (9) conflicts of interest statement, and (10) use of animals with comorbidities.

For meta-analysis, we recorded arterial diameter for intervention and control groups as a percentage from baseline (mean values and a measure of variance with the number of animals per group). Where a single control group was used for multiple treatment groups, we adjusted the size of the control group entered into the meta-analysis by dividing the size of the control group by the number of treatment groups served (43).

Two of the nine publications did not report quantitative data in their text, only presenting graphed data (26, 32). Mean values with SEM were estimated from the graphs of these studies using Universal Desktop Ruler for Windows.

### Statistical Analysis

We performed meta-analysis using normalized mean difference with a random effects model (43). We used univariate meta-regression to explore associations of animal species, sex, strain, and quality issues. We used meta-regression of transformed data using a three-component cubic spline to assess dose–response. Where multiple experiments were performed in the same publication we treated these as separate studies (43). All *in vivo* experiments investigating the effect of αCGRP in animal models of SAH where outcome was reported as a change in arterial diameter were included. We also included experiments which examined *in vivo* SAH and αCGRP administration with postmortem *in vitro* measurements of artery diameter. Where one study administered different doses of αCGRP to separate groups of animals but had a shared control group we treated these as separate experiments and divided the number of controls between the experiments as per Vesterinen et al. (43). Results are presented as the mean ± SE unless otherwise stated. Statistical analysis was performed with STATA 14 (StataCorp LP).

## Results

**Figure 1:**
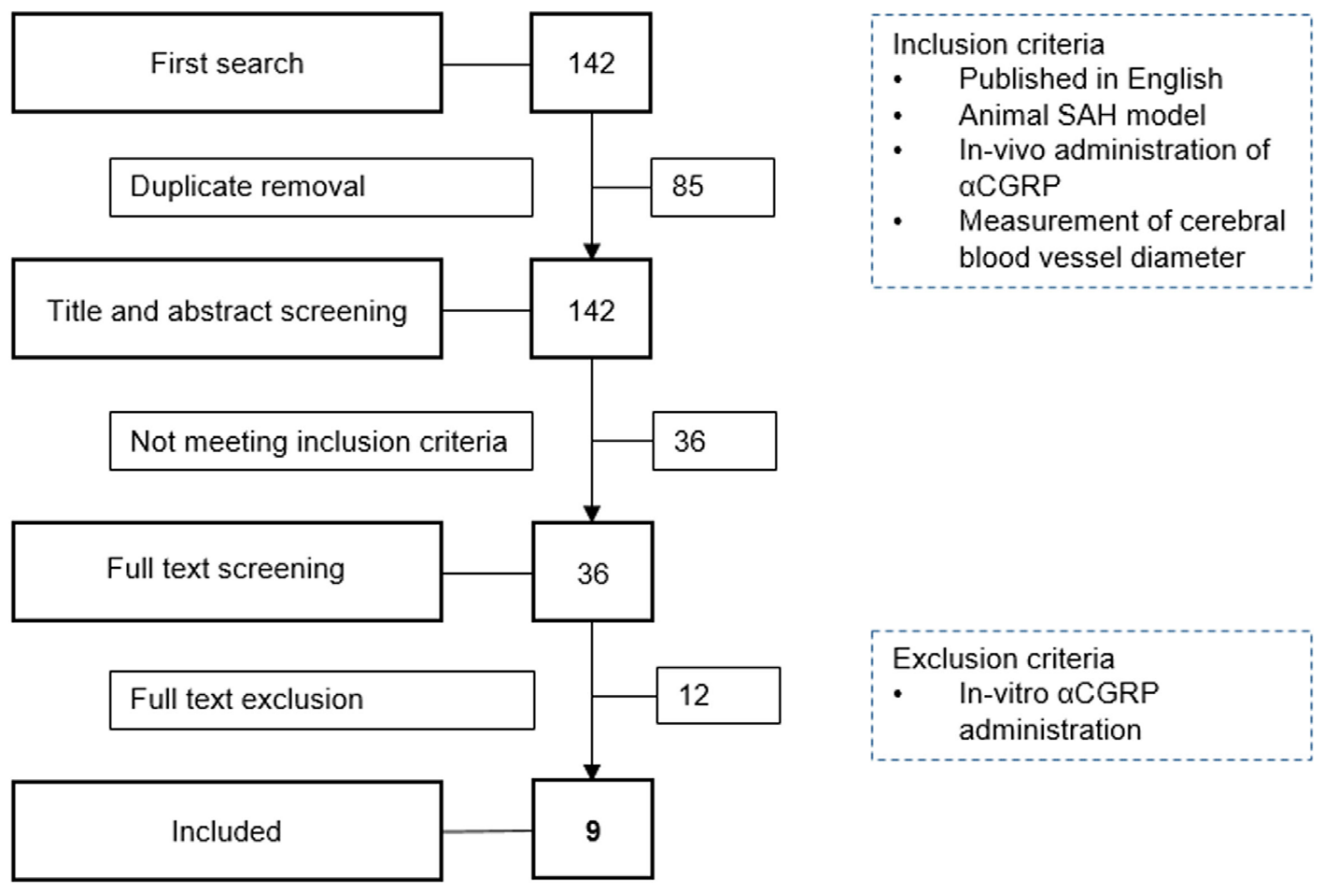
Flowchart of study selection. SAH, subarachnoid hemorrhage; αCGRP, alpha calcitonin gene-related peptide.

We identified 142 publications from our initial search. After combining MEDLINE and EMBASE results and deleting duplicates, 57 publications remained. Titles and abstracts were then screened for eligibility by two authors resulting in 21 publications investigating αCGRP and cerebral vessel narrowing (17–37). After examining the full papers of these abstracts and removing those with *in vitro* αCGRP administration (*n* = 12) and those which lacked SAH models, nine eligible publications remained (17, 21, 22, 26, 27, 32, 35–37). The nine publications included in the review were published between 1989 and 2013 (median year 1996). From the 9 publications, 20 experiments were included in meta-analysis (Figure 1).

### Characteristics of Studies

The total number of animals included in analysis was 251, and the median number of animals used per experiment was 9 [interquartile range (IQR) 6–14]. Twelve experiments used rabbits (*n* = 156, 62% of all animals). Four experiments used New Zealand White, and eight experiments used Japanese White. Five experiments used mongrel dogs (*n* = 45, 18% of all animals). Two experiments used rats (*n* = 40, 16% of all animals). One used Wistar rats, and the other used Sprague Dawley rats. The remaining experiment used *Macaca fascicularis* (*n* = 10, 4% of all animals). The median number of study quality checklist items scored was 4 (IQR 2–6). No studies used animals with comorbidities; reported a statement of potential conflicts of interest or stated an *a priori* sample size calculation. 40% of studies reported control of body temperature. 20% described a randomized treatment allocation, and 45% reported allocation concealment. Half of the experiments used a blinded assessment of outcome, and half used an anesthetic agent other than ketamine. All studies were published in peer-reviewed journals, and 70% reported compliance with local animal welfare guidelines. In 16/20 (80%) of the experiments, SAH was induced by autologous blood injection by either single or double injection methods, the remainder were induced with blood clot placement. Further study characteristics are presented in Tables 1 and 2.

**Table 1:** Study characteristics.

Publications, no.	9
Experiments, no.	20
Animals, no.	251
Median number of animals per experiment, median (IQR)	9 (6–14)
Species, no. (%) of experiments	
Rabbit	12 (60)
Dog	5 (25)
Rat	2 (10)
Monkey	1 (5)
SAH induction, no. (%) of experiments	
Autologous single injection method	12 (60)
Autologous double injection method	7 (35)
Autologous blood clot placement	1 (5)
Vessel examined	
Basilar artery	19 (95)
MCA, ICA, and ACA	1 (5)
Method of visualizing vessel diameter	
Angiography	16 (80)
*In vitro* sections	3 (15)
Direct *in vivo* visualization	1 (5)
Preparation of αCGRP	
CGRP in solution	13 (65)
Gene transfer	4 (20)
Slow-release tablet	3 (15)
Timing of αCGRP administration	
Before SAH	3 (15)
<1 day after SAH	1 (5)
1–3 days after SAH	16 (80)
Time of outcome assessment after administration of CGRP	
<1 h	4 (20)
1–3 days	12 (60)
5–7 days	4 (20)
Study quality [median (IQR)]	4 (2–6)

*IQR, interquartile range; SAH, subarachnoid hemorrhage; αCGRP, alpha calcitonin gene-related peptide; MCA, middle cerebral artery; ACA, anterior cerebral artery; ICA, internal carotid artery*.

**Table 2:** Study characteristics 2.

Reference	Animal	Model	*n* (Control)	Experiments	Delivery route	Delivery time	Time of assessment	Dose	Assessment (diameter)	QS
Nozaki et al. (16, 26)	Dog	SI and DI	6, 5, 5, 6 (11)	4	IC injection	Days 3 and 7	5 min to 24 h postinjection	10–10 to 10–12 mol/kg	BA, % of baseline	1
Toshima et al. (36)	Rabbit	SI	7, 10 (17)	2	IC injection	Day 2	2 h after injection	100 ng/kg/min	BA, micrometers	2
Inoue et al. (22)	Monkey	Blood clot	5 (5)	1	IC tablet	Day 0	Days 0, 7, and 14	400 μg	ICA, MCA, ACA, % of baseline	5
Ahmad et al. (17)	Rabbit	SI	7, 8 (7)	2	IC tablet	Day 2	Days 2–6	24 and 153 μg	BA, % of baseline	4
Imaizumi et al. (21)	Rabbit	SI	7, 7, 5, 5, 5, 5 (36)	6	IC injection	Day 3	Pre-SAH to 24 h post-delivery	10–10 to 10–12 mol/kg	BA, % of baseline	6
Toyoda et al. (37, 45)	Rabbit	SI	8, 8 (14)	2	Gene transfer	5 days before SAH	Days 0 and 2	2 nmol/l	BA, % of control	2
Satoh et al. (27)	Dog	DI	6 (6)	1	Gene transfer	Day 2	Day 7	420 pmol/l	BA, % of mean baseline	6
Sun et al. (32)	Rat	DI	5 (5)	1	Intranasal	Day 3	Day 3	1 μg	BA, micrometers of baseline	4
Tian et al. (35)	Rat	DI	15 (15)	1	Gene transfer	Day 3	Day 7	35.4 ng/l	BA, % of control	4

*SAH, subarachnoid hemorrhage; Model, model of SAH; *n*, number of animals in treatment groups (number of animals in control group); QS, quality score; SI, single injection method; DI, double injection method; IC, intracisternal; BA, basilar artery; MCA, middle cerebral artery; ACA, anterior cerebral artery; ICA, internal carotid artery*.

### Treatment Effect

**Figure 2:**
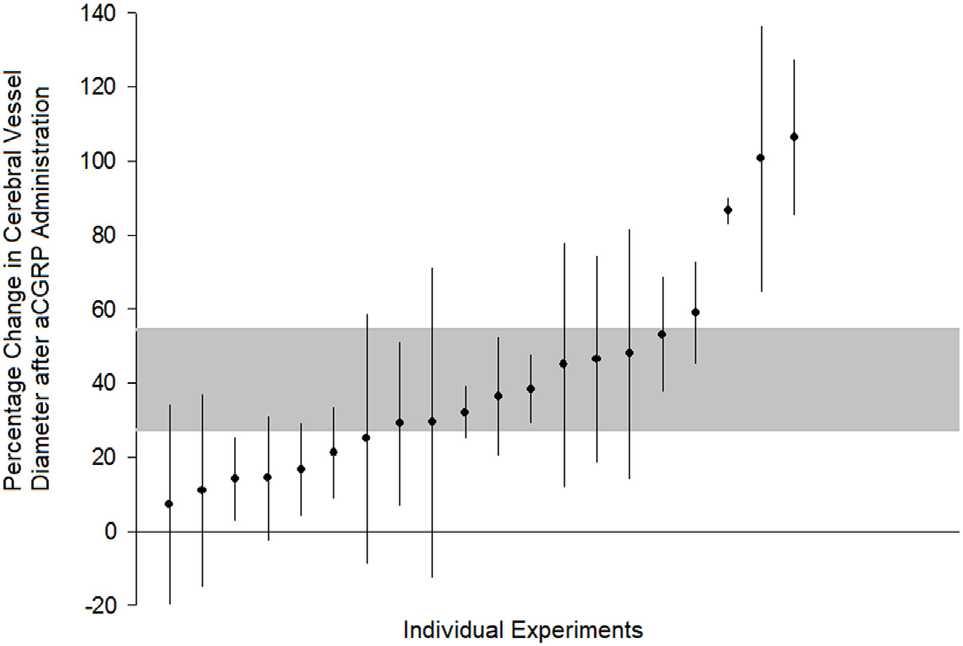
Comparison of individual experiments ranked according to the effect of alpha calcitonin gene-related peptide on change in cerebral vessel diameter. Vertical error bars represent 95% confidence intervals for the individual estimates while the gray area represents the 95% interval for the grouped (all studies) estimate. Percentage change is in comparison to post-subarachnoid hemorrhage diameter or control values, depending upon how the original publication reported results.

**Figure 3:**
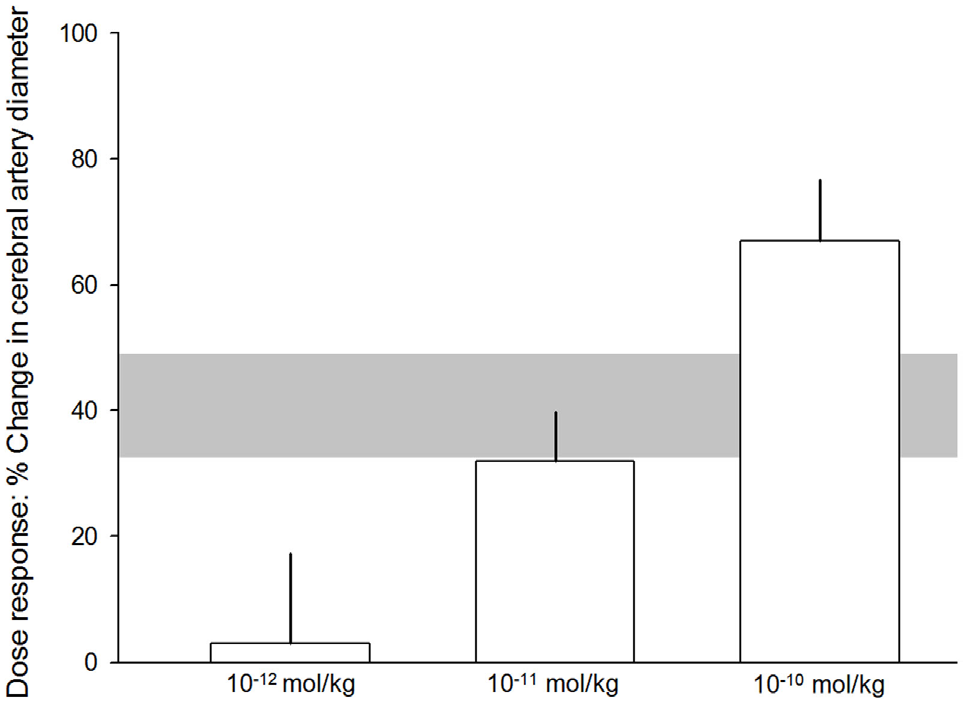
Dose–response relationship. 10^−12^ mol/kg of alpha calcitonin gene-related peptide (αCGRP), 3.1 ± 14.2%. 10^−11^ mol/kg αCGRP, 32 ± 7.8%. 10^−10^ mol/kg αCGRP, 67 ± 9.7%. The error bars represent SE while the shaded gray area represents the SE of the grouped (all experiments with single doses) estimate. Differences were statistically significant (*p* < 0.05).

All 21 publications reporting *in vivo* and *in vitro* experiments demonstrated a dilation of cerebral arteries after αCGRP administration. Of the 20 eligible *in vivo* experiments (taken from the nine eligible publications) included in meta-analysis, there was a 40.8 ± 8.2% increase in cerebral vessel diameter in the αCGRP group compared with controls (*p* < 0.0005, 95% CI 23.7–57.9, *I*^2^ 96%, Figure 2). There was also a significant dose–response to αCGRP in the 10 experiments, which administered a single dose into the cerebroventricular system (Figure 3).

**Figure 4:**
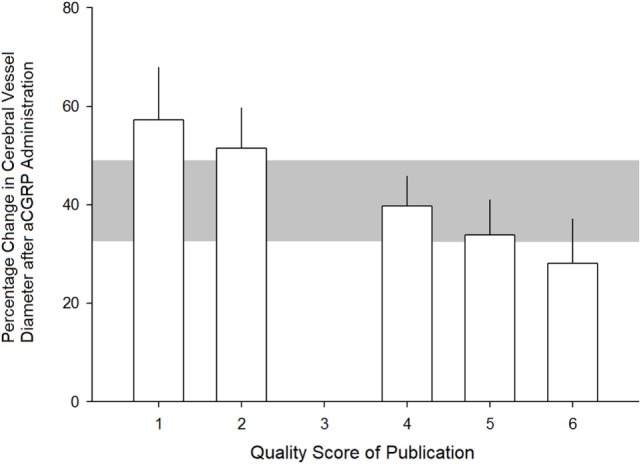
Relationship between quality score of studies and estimated efficacy of alpha calcitonin gene-related peptide at dilating cerebral arteries. See Section “Methods” for quality scoring criteria. Error bars represent SE while the gray area represents the grouped (all studies) estimate. Differences were not statistically significant (*p* > 0.05).

The effect size tended to be lower in studies that reported randomization, blinded assessment of outcome, blinded induction of SAH, and use of an anesthetic agent without intrinsic neuroprotective properties. However, none of these observations reached statistical significance. There was also a trend toward lower effect size in studies reporting compliance with more quality checklist items. This ranged from 57.3 ± 10.7% (*p* < 0.05) from experiments with a quality score (QS) of 1 to 28.1 ± 9.1% (*p* < 0.01) from experiments with a QS of 6 (see Figure 4). There was no statistically significant difference in treatment effect between species.

### Neurological Outcome and Adverse Effects

Four studies reported an effect on neurological outcome after αCGRP administration (17, 21, 22, 35). The standardized mean difference was 1.31 (95% CI −0.49 to 3.12, *Q* 40.5, *n* = 65 animals) in favor of αCGRP. Tian et al. reported neurological outcome based on a comprehensive scoring system (0–48, 0 = best score, 48 = worst score) measured three times daily and based upon the assessment of four functions, which has been used elsewhere (35, 44). The mean neurological outcome on day 7 for the αCGRP group was significantly better than for the control group (10.67 ± 1.16 versus 22.33 ± 2.08, respectively, *p* < 0.001). Imaizumi et al. assessed neurological outcome based on food intake and a slope tolerance test on days 2, 3, and 4 post-SAH (21). No significant difference was found between the αCGRP and control groups for either assessment. Inoue et al. reported no significant difference in food intake, observable hemiparesis, consciousness disturbance or response to stimulation between the αCGRP and control groups (22). Ahmad et al. reported neurological outcome from grade I (normal) to grade III (unable to stand and presented abnormal posture) in addition to performing a slope tolerance test. Two rabbits in the control group were grade II (slow in response but able to walk) and III, respectively, all other rabbits were normal, and there was no statistical difference between the groups in the slope tolerance test. In all studies where it was measured, food intake was decreased after SAH, but there was no significant difference between the αCGRP and control groups. Inoue et al. noted a significant decrease in weight in their αCGRP group compared with the control group at day 14, but note no other adverse effects and were unable to explain this change in weight.

Eight publications reported physiological parameters that might be associated with adverse events. There was no significant difference in systemic arterial pressures or arterial blood gas results between the aCGRP or control groups. Imaizumi et al. found that all animals tended to have an increased respiratory rate for approximately 6 h after intrathecal injection of either αCGRP or vehicle and demonstrated a high blood pH and low pCO_2_, but again no difference between the groups (21). Both Nozaki et al. (16) and Toshima et al. (36) demonstrated a decrease in mean arterial blood pressure when αCGRP was administered intravenously, which was not seen by intrathecal administration. The study by Toshima et al. demonstrates a marked decrease in mean arterial blood pressure following intravenous administration of αCGRP which is not seen with intracisternal administration (~70 versus ~40 mmHg at 30 min after αCGRP administration).

## Discussion

Intrathecal administration of αCGRP dilates cerebral arteries in a dose-dependent manner in animal models and appears to be associated with fewer systemic effects than intravenous administration, chiefly the avoidance of hypotension. Furthermore, the effect of αCGRP on cerebral arteries appears to be more pronounced in the context of SAH, possibly because sensitivity of the artery to αCGRP may be greater owing to the depletion of endogenous αCGRP after SAH (30, 45). Alternatively, it may be that these arteries have a higher capacity for dilation after being constricted following SAH. In addition to the decreased systemic side effects seen by intrathecal administration, this route exposes αCGRP to the abluminal side of the blood vessel wall in a way more akin to its endogenous action. αCGRP is able to dilate cerebral arteries independently of endothelial cells, which are morphologically damaged after SAH and so avoids a problem associated with endothelin antagonists (46, 47).

Other effects of αCGRP were also reported. Sun et al. observed a reduction in cortical cell death, decreased endothelial death and upregulated vascular endothelial growth factor with evidence of angiogenesis after αCGRP administration (32).

There is robust evidence that αCGRP dilates cerebral blood vessels after SAH in animal models, and there is a clear association between DCI and cerebral vessel narrowing in humans. However, it remains unclear whether the association between cerebral vessel narrowing and DCI is causative. Etminan et al. noted in their systematic review and meta-analysis (4,235 patients) that pharmaceutical interventions have decreased the incidence of radiographic vasospasm (decreased cerebral artery diameter on angiography or increased flow velocity on transcranial Doppler) without decreasing poor clinical outcomes (48). The authors note that methodological problems, inadequate sample sizes, insensitivity of clinical outcome measures and mechanisms other than vasospasm that also contribute to poor outcomes could explain the dissociation between vasospasm and clinical outcome in their review. In contrast to these findings, Kimball et al. in a systematic review found that 24 of 27 publications (1,028 patients) reporting the use of transluminal balloon angioplasty noted an improvement in vessel diameter and neurological deficits (49). This review also included mostly small, low-quality studies (based upon the GRADE classification system) (50).

The CONSCIOUS trials investigated the effect of the endothelin-receptor antagonist (ERA), Clazosentan, in the treatment of DCI after SAH (51, 52). Clazosentan was found to produce a dose-dependent reduction in angiographic vasospasm but no significant effect on morbidity or mortality. Endothelin is involved in the regulation of a large variety of organ functions apart from its vasoconstrictor function. It may be that Clazosentan successfully reduced arterial narrowing but also inhibited endothelin’s organ regulatory functions masking any improvement due to improved cerebral diameter. Laban et al., in their systematic review of experimental SAH studies of ERAs, demonstrated a 54% improvement in vessel diameter after administration but no significant effect on mortality and no studies reported effects on functional outcomes (53). The authors concluded that there was no neurobehavioral data to support progression from preclinical to clinical trials for ERAs. In contrast to ERAs, some of the experiments in this review did examine neurobehavioral scores. While there is not a large amount of data, there is a positive signal consistent with a substantial effect. Similarly, a non-statistically significant improvement in outcome for the CGRP group was seen in the European CGRP in SAH trial (RR 0.88, CI 0.60–1.28). Johnston et al. did observe a statistically significant 88.9% treatment preference for CGRP versus placebo in their small study (38, 39). Therefore, although we do not think there is sufficient evidence to support progression directly to a Phase III trial we do think progression to a Phase I trial is appropriate.

### Limitations of Studies

There were no female-only experiments and the majority of experiments in this systematic review used the single hemorrhage model of SAH (60%). The other forms were double injection and clot placement. Animals rarely develop a vessel narrowing-related ischemic neurological deficit from any of these methods. Megyesi et al. note that this is probably because animal brains have a plentiful collateral blood supply (54). While this is probably irrelevant for measuring the effect of αCGRP on vessel diameter, it becomes more problematic when trying to assess neurological outcomes. Another translational problem arises from the times of administration of αCGRP and assessment of neurobehavioral outcomes. In humans, DCI is said to occur most commonly between days 3 and 10 and cerebral vessel narrowing is maximal between days 6 and 10 after ictus (12). The experiments assessed in this review administered αCGRP before and up to 3 days after SAH and assessed the response within hours to one week after administration (Table 1). The authors also note that the best model of vasospasm seems to be the primate model in which a blood clot is placed around a large cerebral vessel (54). Only one of the studies we analyzed uses this model. More recently, Titova et al. in their systematic review state that dog models of SAH are considered superior and that the ability of murine models to reflect human vasospasm is disputed (55).

One of the challenges of translating these animal data and methods to human trials is the invasive nature of the intrathecal route. Previous human trials with intravenous administration of αCGRP have used a continuous infusion owing to the short half-life of αCGPR in the systemic circulation (approximately 7–10 min) (56). However, when administered into the cerebrospinal fluid the effects of αCGRP have continued for 4–6 h (21, 26). Therefore, a continuous infusion of αCGRP into the cerebral spinal fluid may not be necessary. Furthermore, Toyoda et al. (37) and Sun et al. (32) demonstrate novel approaches to administering αCGRP, one *via* gene transfer and the other by intranasal delivery. If intraventricular administration of αCGRP in humans ameliorates cerebral vessel narrowing and avoids the adverse effects seen with intravenous delivery, both gene transfer and intranasal delivery offer potential alternatives without the difficulties associated with an intraventricular drain.

## Conclusion

We demonstrate a significant dilation of cerebral arteries after αCGRP administration in animal models of SAH. However, there is insufficient animal data to determine the effect of αCGRP on neurobehavioral outcomes after SAH. We recommend that any future experimental studies investigating the effect of αCGRP in SAH include neurobehavioral scores as an outcome measure. The dilatory effect of αCGRP appears augmented after SAH, and there is some evidence that systemic effects of αCGRP are lessened by intrathecal administration compared with intravenous administration.

## Author Contributions

LF and CB conducted independent literature searches and performed dual data entry. LF wrote the manuscript. PA and MM edited the manuscript and provided input into review design. MM performed statistical analysis.

## Conflict of Interest Statement

LF and PA have previously applied to the Medical Research Council for funding for a clinical trial involving the administration of αCGRP to patients who have suffered an aSAH.
